# Metabolism dysregulation induces a specific lipid signature of nonalcoholic steatohepatitis in patients

**DOI:** 10.1038/srep46658

**Published:** 2017-04-24

**Authors:** Franck Chiappini, Audrey Coilly, Hanane Kadar, Philippe Gual, Albert Tran, Christophe Desterke, Didier Samuel, Jean-Charles Duclos-Vallée, David Touboul, Justine Bertrand-Michel, Alain Brunelle, Catherine Guettier, François Le Naour

**Affiliations:** 1 Inserm, Unité 1193, Villejuif, F-94800, France; 2 Univ Paris-Sud, UMR-S1193, Villejuif, F-94800, France; 3 DHU Hepatinov, Villejuif, F-94800, France; 4 AP-HP, Hôpital Paul-Brousse, Centre Hépato-Biliaire, Villejuif, F-94800, France; 5 Institut de Chimie des Substances Naturelles, CNRS UPR 2301, Univ. Paris-Sud, Université Paris-Saclay, F-91198 Gif-Sur-Yvette, France; 6 Inserm, Unité 1065, Nice, F-06204, France; 7 University of Nice-Sophia-Antipolis, Nice, F-06204, France; 8 Centre Hospitalier Universitaire de Nice, Hôpital L’Archet, Nice Cedex 3, F-06202, France; 9 Inserm, US33, Villejuif, F-94800, France; 10 Univ Paris-Sud, US33, Villejuif, F-94800, France; 11 MetaToul-Lipidomic Facility, MetaboHUB, Inserm UMR1048, Toulouse, F-31432, France; 12 AP-HP, Hôpital du Kremlin-Bicêtre, Service d’Anatomopathologie, Le Kremlin-Bicêtre, F-94275, France

## Abstract

Nonalcoholic steatohepatitis (NASH) is a condition which can progress to cirrhosis and hepatocellular carcinoma. Markers for NASH diagnosis are still lacking. We performed a comprehensive lipidomic analysis on human liver biopsies including normal liver, nonalcoholic fatty liver and NASH. Random forests-based machine learning approach allowed characterizing a signature of 32 lipids discriminating NASH with 100% sensitivity and specificity. Furthermore, we validated this signature in an independent group of NASH patients. Then, metabolism dysregulations were investigated in both patients and murine models. Alterations of elongase and desaturase activities were observed along the fatty acid synthesis pathway. The decreased activity of the desaturase FADS1 appeared as a bottleneck, leading upstream to an accumulation of fatty acids and downstream to a deficiency of long-chain fatty acids resulting to impaired phospholipid synthesis. In NASH, mass spectrometry imaging on tissue section revealed the spreading into the hepatic parenchyma of selectively accumulated fatty acids. Such lipids constituted a highly toxic mixture to human hepatocytes. In conclusion, this study characterized a specific and sensitive lipid signature of NASH and positioned FADS1 as a significant player in accumulating toxic lipids during NASH progression.

Nonalcoholic fatty liver disease (NAFLD) is a pathological condition involving a broad spectrum of lesions ranging from nonalcoholic fatty liver (NAFL) so-called steatosis to nonalcoholic steatohepatitis (NASH). It has been established that NASH may progress to hepatic fibrosis, cirrhosis and hepatocellular carcinoma^1^,^2^. NAFLD is a systemic disease associated with obesity, insulin resistance, type 2 diabetes mellitus and metabolic syndrome^3^. In addition, NAFLD can also be found in non-obese individuals and its prevalence can range from 3.5% to 27% in lean individuals^4^. Therefore, the dramatic increased incidence of NAFLD makes it the most common cause of chronic liver diseases and a major public health problem worldwide^5^,^6^.

The hallmark of fatty liver disease is an intracellular accumulation of lipids, and particularly triglycerides, which causes the formation of lipid droplets in hepatocytes. This accumulation results from an imbalance between the uptake, synthesis, export and oxidation of fatty acids^7^. Fatty liver is a reversible and asymptomatic lesion that has long been considered as benign. However, it is now admitted that fatty liver is a precursor for steatohepatitis defined by the presence of steatosis, ballooning of hepatocytes, Mallory’s bodies and lobular inflammation with infiltrated macrophages and leukocytes on liver histology^8^,^9^. In line with this assumption, we have demonstrated using transcriptomic analysis that genes involved in inflammatory processes were significantly up-regulated in patients with bland steatosis^10^ leading to the main idea that NAFL is not so benign^11^, and suggesting that lipids themselves may trigger inflammation. More precisely, it has been reported that some lipid species such as saturated fatty acids, phospholipids and disturbances in ceramide-signaling or cholesterol content exhibit pro-inflammatory and pro-apoptotic properties^7^,^12^,^13^,^14^. Therefore the progression of fatty liver to NASH may be related to the lipid composition. A few studies in human have focused on comprehensive hepatic lipidomic analysis in order to identify the lipids involved in NAFLD^15^,^16^,^17^. These studies revealed changes to the homeostasis of triglycerides, cholesterol, phospholipids and long-chain fatty acids (LCFA) in that context. However, none of these studies was able to characterize a specific lipid signature and mechanism for NASH. Therefore, identifying lipids with potential toxicity related to the progression of NASH is still an unmet need.

In this study, we have performed a comparative lipidomic analysis on human liver biopsies from patients with NAFL or NASH. We employed the unbiased mathematical approach that we recently implemented on animal models. Indeed, we have demonstrated that a random forests-based mathematical approach was enabled to characterize hepatic lipid signature specific to NASH in murine models^18^. In patients with NASH, we showed here the universal hallmark of the lipid signature of NASH that was related to alterations of the metabolic pathway involved in the synthesis of LCFA and very long-chain fatty acids (VLCFA). Finally, we demonstrated that lipids selectively accumulated in the context of NASH constituted a mixture highly toxic to human hepatic cells.

## Results

### Lipidomic and machine learning analysis revealed a lipid signature of NAFLD

A quantitative lipidomic analysis was performed in order to identify lipids discriminating the pathological statuses of the liver. The study was realized on 61 liver biopsies including normal livers as controls (n = 7), nonalcoholic fatty livers (NAFL, n = 39) and nonalcoholic steatohepatitis (NASH, n = 15), from the same hospital (Hôpital Paul Brousse, Villejuif, France) (Table 1). Lipids were extracted from the liver tissue and further identified by gas phase or liquid phase chromatography coupled to mass spectrometry (GC/LC-MS). This led to the identification and quantification of 104 lipid species such as cholesterol, cholesteryl esters (CE, n = 3), one diacylglycerol (DG), triglycerides (TG, n = 5), fatty acids (n = 21), ceramides (Cer, n = 4), phosphatidylcholines (PC, n = 18), phosphatidylethanolamines (PE, n = 16), phosphatidylinositols (PI, n = 14), phosphatidylserines (PS, n = 11) and sphingomyelins (SM, n = 11).

First, we sought to distinguish different grades of steatosis among the 39 liver biopsies from NAFL. Thus, in order to avoid any bias related to an imbalance and to assure a homogenous repartition of patients between NAFL groups, we employed the classification and regression trees (CART) analysis. Indeed, we have previously demonstrated by using this approach that the total amount of triglycerides (TG) allowed the unbiased discrimination of the grade of steatosis^19^ much better than histological examination usually considered as the “gold standard”^20^. In the present study, CART approach led to individualize 3 groups of nonalcoholic fatty livers based on the total amount of TG, namely NAFL1 (41.7 < TG < 220 nmol/mg of protein; n = 9), NAFL2 (220 < TG < 465.5 nmol/mg of protein; n = 12) and NAFL3 (TG > 465.5 nmol/mg of protein; n = 18) (see Supplemental Fig. S1). We confirmed whether the number of patients per group was suitable for further statistical analyses. We performed a statistical parametric test by multivariate analysis of variance (MANOVA) that showed a minimum number of 54 patients with an average of 9 patients per group. Our study groups that enrolled a total of 61 patients was in the upper range compared to other studies published in the field (Table 1)^15^,^21^,^22^,^23^,^24^.

**Figure 1:**
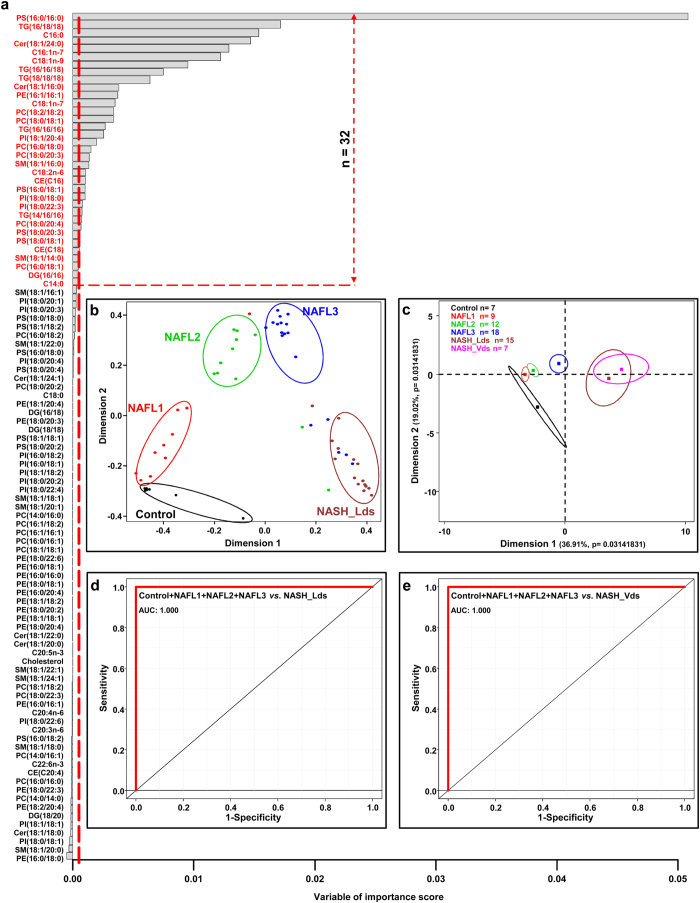
Selected lipids using random forests analysis discriminate NASH patients. (**a**) Data are represented as a bar plot of a matrix with 104 lipids (column) analyzed by mass spectrometry and ordered based on their variable of importance score. The threshold in the x-axis is calculated as the absolute value of the less abundant lipid represented as vertical red dot line. Discriminant lipids (n = 32) are those over the horizontal red dot line. Random forests analysis was run with the 5 groups of patients. (**b**) Multidimensional scaling plot discriminating NASH_Lds group from Control, NAFL1, NAFL2 and NAFL3 groups based on random forests results. (**c**) Principal component analysis based on the 32 lipids identified discriminating specifically NASH patients (NASH_Lds). Lines are the ellipses centered to the mean (colored squares) representing 95% interval confidence, and p the probability associated with the F- test of the analysis of variance along the axes. ROC curves based on the 32 lipids combined comparing (**d**) NAFLD groups of patients (*i.e*. Control + NAFL1 + NAFL + NAFL3) and NASH group (NASH_Lds) from Paul Brousse Hospital, and (**e**) NASH group (NASH_Vds) from L’cc. • Control n = 7; 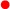 NAFL1 n = 9; 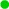 NAFL2 n = 12; 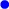 NAFL3 n = 18; 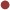 NASH_Lds n = 15, 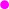 NASH_Vds n = 7. AUC: Area under the curve; CE: cholesteryl ester; Cer: Ceramides; DG: diacylglycerols; NAFL: nonalcoholic fatty liver; NASH: nonalcoholic steatohepatitis; PC: Phosphatidylcholines; PE: Phosphatidylethanolamines; PI: Phosphatidylinositols; PS: Phosphatidylserines; PV: predictive value; SM: Sphingomyelins, TG: triglycerides.

Investigations were further conducted using a random forest-based machine learning approach. This unbiased statistical analysis allows comparing several groups -each exhibiting hundreds variables- thus leading to characterize the predictor variables of a given status^18^,^25^. Studies were performed on the whole set of data corresponding to 104 variables from the 61 liver biopsies distributed in 5 groups including normal liver (n = 7), NAFL1 (n = 9), NAFL2 (n = 12), NAFL3 (n = 18) and NASH called “learning data set” (NASH_Lds, n = 15). After calculating the best number of randomly preselected splitting variables (mtry = 28, see Supplemental Fig. S2a), random forests analysis led to the characterization of a signature constituted by 32 lipids based on mean decreased accuracy (MDA) and mean decreased Giny (MDG) scores (Fig. 1a, see Supplemental Fig. S2b). Such a signature allowed discriminating the 5 groups from each other (Fig. 1b) and was associated to out-of-bag (OOB) estimate of error rate of 14.75% (see Supplemental Fig. S2b). Indeed, normal livers were distinguished from low level of steatosis NAFL1 along the first dimension that is related to the highest variance. The three groups NAFL1, NAFL2 and NAFL3 were well discriminated (Fig. 1b). Finally, the NASH_Lds patients appeared as a compact group onto the second dimensions of the plot that was completely separated from the other groups (Fig. 1b).

The study was further focused on the validation of the lipid signature of NASH. Additional liver biopsies from 7 patients with NASH called “validation data set” (NASH_Vds) were obtained from another hospital (Hôpital L’Archet, Nice, France). The characteristics of the study groups were summarized in Table 1. Lipids were extracted from these biopsies followed by quantitative lipidomic analysis. Using the signature of the 32 lipids, the projection into the two dimensional plot of the validation group NASH_Vds was superimposed to NASH_Lds (Fig. 1c). Furthermore, receiver operating characteristic (ROC) curve demonstrated that the overall signature of 32 lipids was able to discriminate NASH_Lds (Fig. 1d, see Supplemental_Excel_File_S1) as well as NASH_Vds (Fig. 1e, see Supplemental_Excel_File_S2) from other groups of patients with 100% sensitivity and specificity (Fig. 1d and e).

**Figure 2:**
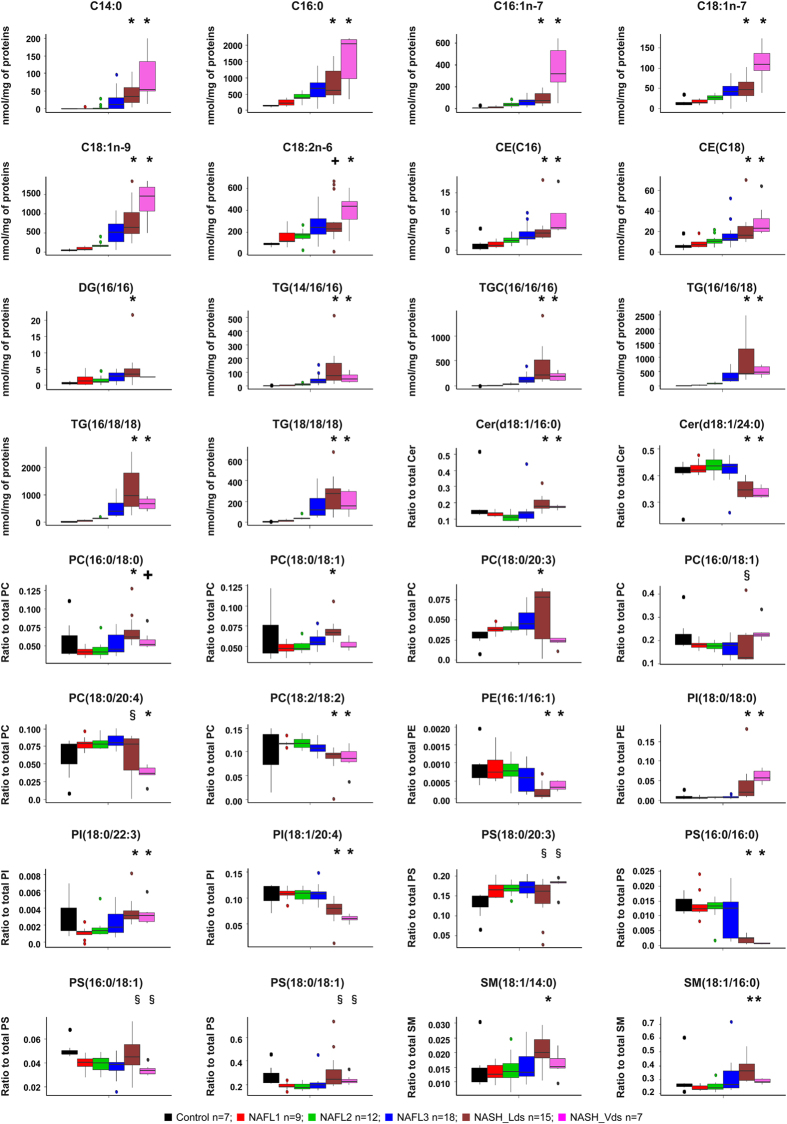
Hepatic levels of the 32 lipids discriminating NASH group based on random forests analysis. Data are represented as boxplot. *p < 0.05, by unpaired *t*-test compared to Control, NAFL1, NAFL2 and NAFL3 groups. ^+^p < 0.05 by unpaired *t*-test compared to Control, NAFL1 and NAFL2 groups. ^**§**^p < 0.05 by unpaired *t*-test compared to Control group. Unpaired *t*-test was done after ANOVA test. ■ Control n = 7; 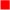 NAFL1 n = 9; 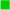 NAFL2 n = 12; 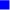 NAFL3 n = 18; 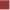 NASH_Lds n = 15; 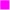 NASH_Vds n = 7. NAFL: nonalcoholic fatty liver; NASH: nonalcoholic steatohepatitis; NASH_Lds: learning dataset; NASH_Vds: validation dataset.

Among the 32 discriminant lipids, 9 lipids were significantly decreased in NASH, mainly ceramides and phospholipids. On the other hand, 23 lipids exhibited a significant increase in NAFL and/or NASH, including 6 fatty acids, 2 cholesteryl esters, 1 diglyceride, 5 triglycerides and 9 phospholipids (Fig. 2).

Altogether, these results demonstrated that the random forests mathematical approach allowed characterizing a lipid signature of the grades of fatty liver diseases discriminating NASH patients with 100% accuracy.

### Lipid signature of NASH was related to dysregulations along fatty acid synthesis pathway

**Figure 3:**
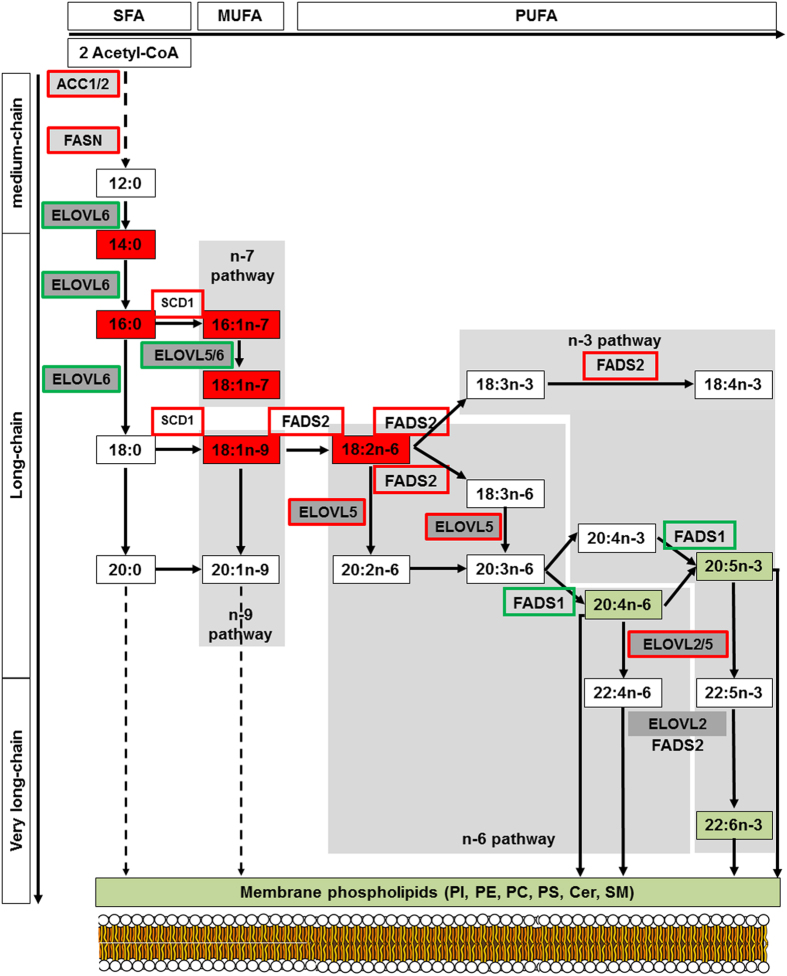
Scheme of short-, long- and very long-chain fatty acid biosynthesis leading to membrane phospholipids synthesis. The long chain saturated fatty acids and unsaturated fatty acids of the n-3, n-6, n-7 and n-9 series can be synthesized from myristic acid (C14:0) and palmitic acid (C16:0) produced by ACC and FASN. Long-chain fatty acids of the n-6 and n-3 series can also be synthesized from precursors obtained from dietary precursors to elongation (ELOVL) and desaturation (FADS) steps as indicated in these pathways. Lipids in red and in green are those found “up” and “down” in our analysis, respectively. Increase in enzyme activities is framed in red whereas a decrease is framed in green. ACC: acetyl-CoA carboxylase; ELOVL: elongase of very long chain fatty acid; FASN: fatty acid synthase; FADS: fatty acid desaturase; SCD: stearoyl-CoA desaturase.

The metabolic pathways deregulated in NASH were investigated. Interestingly, the 6 fatty acids increased in NASH (C14:0, C16:0, C16:1n-7, C18:1n-7, C18:1n-9 and C18:2n-6) belong to the LCFA synthesis pathway (Fig. 3). Thus, investigations were performed on the activity of the enzymes along this metabolic pathway involving elongases and desaturases. The activity of each enzyme was estimated by measuring the ratio between its product and substrate^26^ based on our lipidomic analysis.

**Figure 4:**
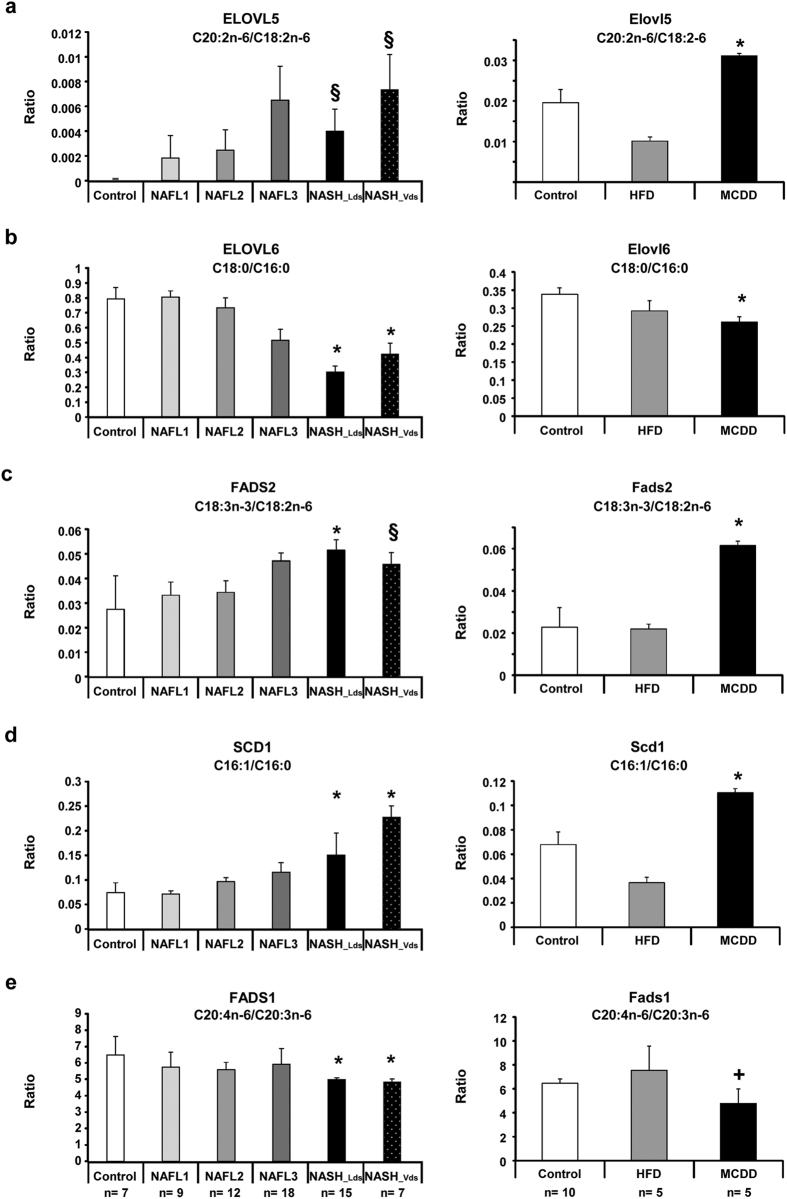
Decrease in ELOVL 6 and FADS1 activities in NASH patients and mouse feeding a methionine-choline deficient diet. In order to evaluate enzyme activities, ratio between product to precursor of each reaction has been used in human groups (left panel) and mouse models (right panel). (**a**) Evaluation of ELOVL5 activity using C20:2n-6 to C18:2n-6 ratio, (**b**) ELOVL6 activity using C18:0 to C16:0 ratio, (**c**) FADS2 activity using C18:3n-3 to C18:2n-6 ratio, (**d**) SCD1 activity using C16:1 to C16:0 ratio and (**e**) FADS1 activity using C20:4n-6 to C20:3n-6 ratio. Data are shown as mean ± SEM. *p < 0.05 by unpaired *t*-test compared to each other groups. +<0.05 by unpaired *t*-test compared to Control and ^§^p < 0.05 by unpaired *t*-test compared to Control. NAFL1 and NAFL2 after ANOVA analysis. Control patients n = 7; NAFL1 n = 9; NAFL2 n = 12; NAFL3 n = 18; NASH_Lds n = 15; NASH_Vds n = 7. Control mice n = 10; HFD n = 5; MCDD n = 5.

First, we focused on the two main elongases named ELOVL (elongation of very long chain fatty acids), ELOVL5 and ELOVL6. ELOVL5 activity was significantly increased (Fig. 4a, Supplemental Fig. S3a), whereas ELOVL6 activity was significantly decreased (Fig. 4b). ELOVL6 is involved in the elongation of lauric acid (C12:0) to stearic acid (C18:0) (Fig. 3). The decrease of ELOVL6 activity in NASH was consistent with the marked increase in LCFA in NASH culminating with the accumulation of myristic acid (C14:0) (Fig. 2). Investigations were further performed on the activity of the desaturases fatty acid desaturase 1 (FADS1), FADS2 and steroyl-CoA desaturase 1 (SCD1). FADS2 and SCD1 activities were increased in accordance with the increase of monounsaturated (C16:1n-7 and C18:1n-9) and polyunsaturated (C18:2n-6) fatty acids in NASH (Fig. 4c and d, Supplemental Fig. S3b and c). In contrast, a significant decrease in FADS1 activity was observed in NASH (Fig. 4e). Furthermore, the estimation of amount of substrate and product on multiple steps suggested that ELOVL6 and FADS1 were limiting enzymes along the metabolic pathway. Indeed, the decreased activity of these two enzymes was driving the global activity of the pathway significantly down in NASH patients (Supplemental Fig. S3d–h). Accordingly, this led to a significant increase in n-6 to n-3 ratio and significant decrease n-3 index in NASH patients (Fig. 3, Supplemental Fig. S3i), which are both markers of inflammatory process during NASH progression.

**Figure 5:**
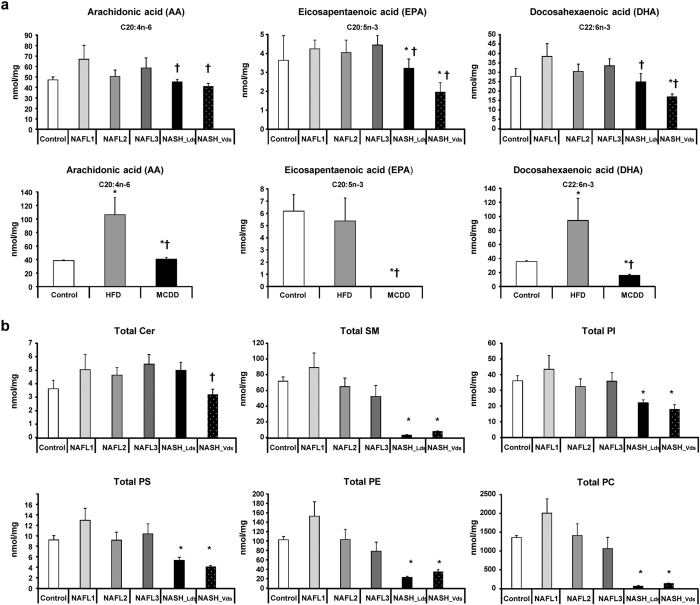
Decrease in eicosanoid precursors in NASH. Hepatic levels of (**a**) arachidonic acid (C20:4n-6), eicosapentanoic acid (C20:5n-3), and docosahexaenoic acid (C22:6n-3) in the patients studied (upper panel) and mouse models (lower panel). (**b**) Total phospholipids in each group of patients. Data are means ± SEM. *p < 0.05, by unpaired *t*-test compared to Control group and ^†^p < 0.05, by unpaired *t*-test compared to NAFL groups after ANOVA analysis. Control n = 7; NAFL1 n = 9; NAFL2 n = 12; NAFL3 n = 18; NASH_Lds n = 15; NASH_Vds n = 7 and Control n = 10; HFD n = 5; MCDD n = 5.

The decrease in FADS1 desaturase activity in NASH may constitute a bottleneck leading to the accumulation of fatty acids upstream. As a consequence, the synthesis of lipids downstream of this enzyme such as the eicosanoid precursors (arachidonic acid (C20:4n-6), eicosapentaenoic acid (C20:5n-3) and docosahexaenoic acid (C22:6n-3)) exhibited a significant decrease in livers of NASH compared to controls (Fig. 5a). It should be noted that eicosanoid precursors are involved in the synthesis of phospholipids. Therefore, the deficiency in the synthesis of polyunsaturated LCFA has to produce a global decrease in phospholipids that was observed in NASH patients (Fig. 5b).

To determine if changes in desaturase and elongase activities were related with their expression levels, liver mRNA gene expressions of *ELOVL5, ELOVL6, FADS1, FADS2* and *SCD1* were investigated by RT-Q-PCR on NASH compared to normal livers and NAFL matched NASH patients. To avoid any variations of mRNA gene expression levels due to the age, gender, BMI and steatosis grade (*i.e*. the amount of lipid content), patients from NAFL2 group were matched to NASH patients (Fig. 2 and see Supplemental Fig. S4). We first verified that the lipid signature in these subgroups of patients was identical to the whole group of patients (Fig. 2 and Supplemental Fig. S4, respectively). *ELOVL5* mRNA expression was slightly but significantly increased in NASH compared to NAFL2 group (Supplemental Fig. S5a) whereas *ELOVL6* mRNA liver expression was significantly decreased in NASH patients (Supplemental Fig. S5b), consistent with low enzyme activity observed in NASH. The gene expression of *FADS2* and *SCD1* were significantly increased in NASH patients (Supplemental Fig. S5c and d) according to the enzyme activities observed. Regarding *FADS1*, liver mRNA gene expression level was similar in NASH compared to control group and only a slight decrease was observed in NASH compared to NAFL2 group (Supplemental Fig. S5e). Studies were further focused on genes related to *de novo* fatty acids synthesis such as sterol regulatory element-binding proteins 1c (*SREBP1c*), fatty acid synthase (*FASN*) and acetyl-CoA carboxylase 1 (*ACC1*) (Fig. 3). As previously reported in human livers^27^,^28^,^29^,^30^,^31^,^32^,^33^, *SREBP1c* expression was significantly decreased in NAFL patients compared to control whereas it was increased in NASH patients compared to NAFL2 (Supplemental Fig. S5f). Interestingly, *FASN* and *ACC1* gene expression levels were significantly decreased in NAFL group but significantly increased in NASH, suggesting that *de novo* fatty acid synthesis may contribute to LCFA accumulation in NASH (Supplemental Fig. S5g and h).

Altogether, these results highlighted the major dysregulation of fatty acid synthesis pathway in NASH. Changes in lipid composition in NASH resulted of the additional effect of increase in *de novo* short-chain fatty acids synthesis and in FADS2 and SCD1 activities associated to a decrease in ELOVL6 and FADS1 activities. These results also positioned FADS1 as a bottleneck leading upstream to the accumulation of LCFA, and downstream to the deficiency in VLCFA and thus in phospholipids synthesis.

### Dysregulations along fatty acid synthesis pathway were confirmed in animal models

The metabolic features observed in human were investigated using animal models. NAFL and NASH can be induced in mice by using specific high-fat diet (HFD) and methionine-choline deficient diet (MCDD), respectively^14^,^34^,^35^,^36^,^37^,^38^. We exploited the lipidomic analysis of these mouse models that we published recently^18^ to confirm the data observed in patients. Interestingly, Elovl5, Fads2 and Scd1 activities were also increased in MCDD mice (Fig. 4a,c and d, respectively). In contrast, Elovl6 and Fads1 activities were decreased (Fig. 4b and e, respectively). The amount of lipids synthesized downstream Fads1 such as arachidonic acid (C20:4n-6), eicosapentaenoic acid (C20:5n-3) and docosahexaenoic acid (C22:6n-3) were dramatically decreased in livers of mice fed MCDD (Fig. 5a).

These observations demonstrated the common metabolic dysregulation along the fatty acid synthesis pathway in both human patients and animal models, leading to the development of NASH.

### Mass spectrometry imaging on tissue section revealed spreading of lipids in NASH

**Figure 6:**
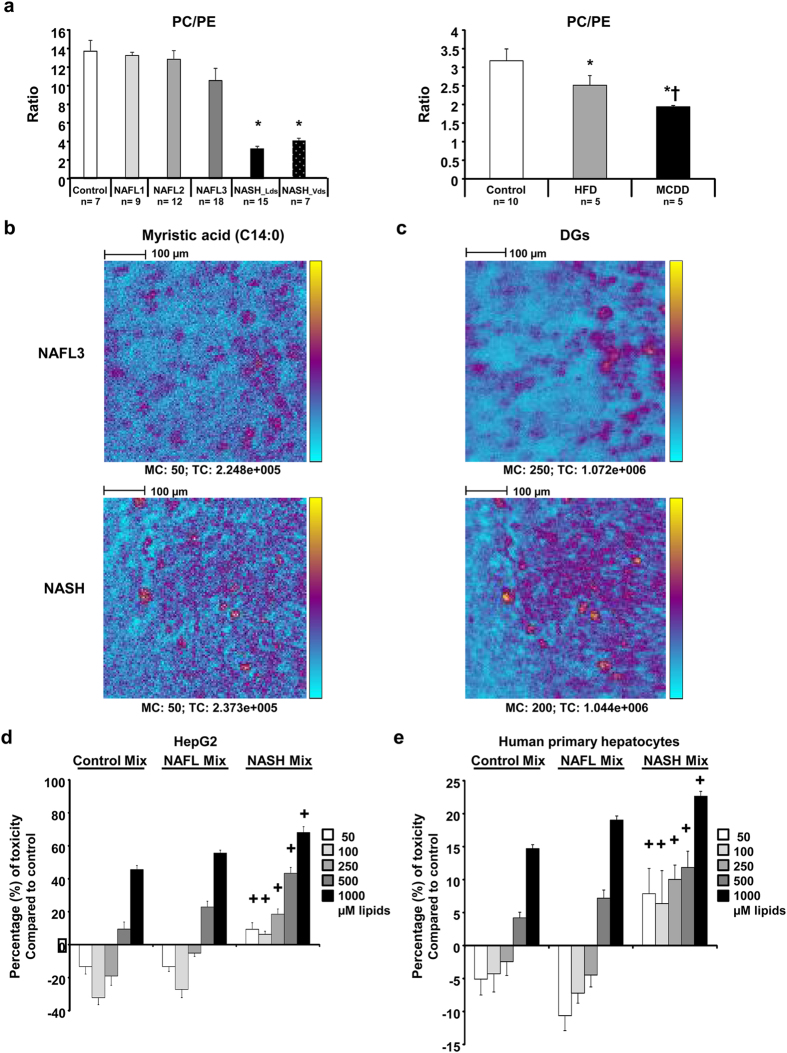
Membrane integrity is disrupted in NASH patients and mice leading to release of lipids in parenchyma. (**a**) Phosphatidylcholine (PC) to phosphatidylethanolamine (PE) ratio in the patients studied (left panel) and mouse models (right panel). (**b**) Myristic acid (C14:0) and (**c**) sum of all diacylglycerols (DG) liver contents in NAFL3 and NASH patients. Both selected patches regarding myristic acid were matched according to the total count (TC). Each patch is representative of the 6–12 patches recorded from central vein to portal triad. Between 2 to 3 slices per sample were processed. Color scale bar with amplitude in number of counts are indicated to the right of each image. Field of view of 500 μm × 500 μm. Scale bars: 100 μm. Data are means ± SEM. *p < 0.05 by unpaired *t*-test compared to Control group and ^†^p < 0.05 by unpaired *t*-test compared to NAFL groups after ANOVA analysis. Control n = 7; NAFL1 n = 9; NAFL2 n = 12; NAFL3 n = 18; NASH_Lds n = 15, NASH_Vds n = 7 and Control n = 20; HFD n = 5; MCDD n = 5 mouse males. (**d**) HepG2 cell and (**e**) human primary hepatocytes treated with lipid mixes at different concentrations. Cells are treated in triplicate during 24 h with 50. 100. 250. 500 and 1000 μM final concentration of lipids with lipid mixes (Control Mix. NAFLD Mix. NASH Mix) based on the percentage of C14:0, C16:0, C16:1n-7, C18:1n-7, and C18:1n-9 found in liver tissues of control. NAFL2/3 and NASH patients. Two independent experiments were done. Data are mean ± SEM. ^+^p < 0.05 by unpaired *t*-test compared to Control Mix and NAFL Mix at the same concentrations after ANOVA test.

A major feature of NASH revealed by our lipidomic analysis was the failure in total phospholipids (Fig. 5). The low amount of phospholipids may impact cellular membranes in which phospholipids are important components. Furthermore, it has been reported that the ratio between phosphatidylcholine (PC) and phosphatidylethanolamine (PE) can be used as a surrogate to assess cell membrane integrity^39^. Investigations were performed in both humans and mouse model thus demonstrating a significant decrease of the PC to PE ratio in NASH (Fig. 6a). These data suggested cell membrane impairments leading to a possible spreading of hepatocyte content into hepatic parenchyma. In order to address the distribution of lipids in the liver tissue, experiments were performed using time-of-flight-secondary ion mass spectrometry (ToF-SIMS) imaging. This approach allows investigating the lipid composition at the subcellular level. By rastering a tissue section, the distribution of lipids can be visualized. Mass spectrometry imaging using ToF-SIMS was performed on tissue sections from patients with NAFLD. The distribution of fatty acids C14:0 as well as C16:0, C18:0, C16:1, C18:1, C18:2 and C20:4 to consolidate data (data not shown) were addressed. The distribution of diacylglycerols (DAG) corresponding mostly to the fragmentation of triglycerides under mass spectrometry analysis was also addressed. In NAFL patients, lipids were accumulated into lipid droplets (Fig. 6b and c). In patients with NASH, the lipids were also accumulated into lipid droplets but an important diffusion was observed into hepatic parenchyma (Fig. 6b and c). It should be noted that the comparison in the lipid repartition between NAFL and NASH was performed from images exhibiting similar amount of the lipid species studied as attested by the total count (TC) values (TC_NAFL3_ = 2.25 × 10^5^ vs TC_NASH_ = 2.37 × 10^5^), thus strengthening a real difference in terms of distribution.

These results suggested a disruption of cell membrane integrity in NASH, most likely due to a defect in phospholipids, leading to a leak and spreading hepatocyte content out into the parenchyma.

### Specific mixture of lipids accumulated in NASH exhibited higher toxicity on hepatocytes

The toxicity of the lipids identified in NASH was addressed. Studies were focused on 5 fatty acids accumulated in NASH and available for cell culture. Toxicity of myristic acid (C14:0), palmitic acid (C16:0), palmitoleic acid (C16:1n-7), vaccenic acid (C18:1n-7) and oleic acid (C18:1n-9) were investigated in cell culture on HepG2 cells and human primary hepatocytes (HPH). Lipotoxicity was first addressed for each individual lipid at various concentrations (50, 100, 250, 500 and 1000 μM). HepG2 cell line and HPH showed different sensitivities to lipids most likely due to differences in the lipid metabolism of such cells^40^. Moreover, the 5 lipids at the highest concentration exhibited toxicity on both HepG2 cells and HPH by triggering 25% to 90% cell death (Supplemental Fig. S7a and b). Toxicity of combined lipids was further investigated. Three mixes corresponding to the composition and proportion of the 5 fatty acids into the normal liver, NAFL2/3 and NASH were composed based on the mean concentrations obtained from our lipidomic analysis, respectively (Table 2). HepG2 cells and HPH were incubated with such mixes at the same final concentrations from 50 μM to 1000 μM. Interestingly, NASH mix was significantly more toxic on both hepatic cells. Furthermore, lipotoxicity of the NASH mix was also observed at low concentration (Fig. 6d and e).

These results demonstrated the potent toxicity of the specific mixture of lipids accumulated in NASH.

## Discussion

The aim of this study was to characterize lipid markers specific to all patients with NASH independently of patient’s background such as mild (between 30 and 35 kg/m^2^), severe (between 35 and 40 kg/m^2^) or morbid (>40 kg/m^2^) obesity associated or not with type II diabetes assessed by the homeostasis model assessment of insulino-resistance (“low-HOMA-IR” or “High-HOMA-IR”), and/or a metabolic syndrome. In order to find universal lipid markers for the NASH, we intentionally not focused in patients’ medications or systemic complications such as type II diabetes and insulin-resistance as well as their polymorphisms or even gender. Difference in female gender distribution between groups of patients was not linked to the progression of the steatohepatitis but due to the selection between both hospitals due to their specificities (*i.e*. liver diseases and liver transplantation *vs* bariatric surgery). Also, the role between genders and steatohepatitis progression is not elucidated yet^8^,^41^,^42^.

Here, we used an innovative unbiased random forests-machine learning statistical analysis^25^ that was never used before to find biomarkers in patients. This approach allowed not being dependent of the size of the patient cohort which is usually a limiting factor when analyzing multiple groups of patients involving hundreds of variables using conventional statistical methods (*e.g*. ANOVA, MANOVA).

For the first time, this study established a lipid signature of nonalcoholic steatohepatitis based on the quantification of 32 lipids. Such complex signature highlighted the major interest of combining a global approach such as lipidomic with an unbiased RF analysis. Indeed, none of the lipids identified allowed by itself the discrimination of NAFL or NASH, but in contrast the overall lipid signature allowed discriminating between control patients, NAFL groups and NASH patients. The robustness of the lipid signature of NASH was underlined by 100% specificity and 100% sensitivity on samples from two independent hospital centers. Indeed, using two independent groups of patients with NASH from these hospitals emphasizes the sturdiness and the universality of the lipid signature of NASH. Moreover, the main difficulty for a pathologist is to discriminate between steatosis and steatohepatitis, especially before the inflammation and hepatocyte degenerations appeared (*i.e*. ballooning and Mallory’s hyaline bodies). Therefore, this specific lipid signature is able to discriminate between both steatosis and steatohepatitis.

Dysregulations of the metabolic pathway involved in synthesis of fatty acids were highlighted in NASH. The major impact of alterations in this metabolic pathway was reinforced by the similar biochemical features (*i.e*. dysregulation of enzyme activities) observed in both human and animal models for this pathology, strengthening the idea that these alterations are universal in NASH. Indeed, we used animal models that developed fatty liver when fed on high fat diet or NASH when fed on methionine choline deficient diet. It should be noted that MCDD mouse model is the most well-known and used model to study NASH^18^,^21^,^34^,^35^,^36^,^37^,^43^. A short period of 5 weeks is enough to develop NASH. Thus, the murine models led to control the environment and to confirm the observations found in humans. Most of the time when NASH is diagnosed in human patients, the disease is already progressing and therefore the main underlying mechanism is blunt by subsequent modifications during the chronic phase. Finally, using mouse models permitted to control food and obesity and demonstrated that the decreased in FADS1 activity in mice fed MCDD and human patients with NASH is not dependent of diet and obesity as demonstrated also by others^44^.

The concomitant increase of saturated and unsaturated LCFA with the significant decrease of polyunsaturated VLCFA resulted from the decreased activity of FADS1. Impairment of FADS1 activity has created a bottleneck leading to the accumulation upstream of fatty acids up to 20 carbons as also described before^45^, but interestingly in our study we found it associated to the NASH. This phenomenon was accentuated by an increase in *de novo* fatty acids synthesis as demonstrated by the increase in mRNA expression levels of *ACC1* and *FASN*. Furthermore, decreased expression and activity of ELOVL6 contributed also to the marked increase of LCFA culminating in NASH with the accumulation of myristic acid (C14:0). Recently, it has been demonstrated that fatty acid metabolism was altered in NASH independent of obesity and diet^44^. Expression and activities of FADS1 (also called delta-5 desaturase, D5D), FADS2 (also called delta-6 desaturase, D6D) and SCD1 were dysregulated. Lower activity of FADS1/D5D was observed^44^ in NASH groups and was independent of diet and obesity as we demonstrated in our study in both patients and murine models. In addition, FADS2 and SCD1 mRNA liver expression were significantly increased in NASH group according to our observations and by others^44^. On the other hand, a consequence of the impaired expression and activity of FADS1 was the extremely low amount of polyunsaturated LCFA added to the hepatic imbalance between n-6 and n-3 levels. Indeed, our results demonstrated that n-6 to n-3 ratio was significantly increased, associated with a significant decrease in n-3 index in livers of both patients with NASH and MCDD mice, according to recent studies^26^,^45^,^46^,^47^. Such metabolic alterations may generate broad effects since LCFA represent substrates for the synthesis of eicosanoids and phospholipids, thus impacting the properties of membranes. LCFA also serve as substrate precursors for the biosynthesis of lipid signaling molecules with pro-inflammatory properties^48^,^49^,^50^. Therefore, the current study strengthened the central role of FADS1 in lipid homeostasis and positioned this desaturase as a major player in NASH^45^.

Thus, due to the impairment in FADS1 activity, the deficiency in phospholipid synthesis can damage the cellular membranes in which phospholipids are major components. Indeed, half of the phospholipids from the signature were significantly down in NASH patients Furthermore, we have shown that a surrogate of membrane integrity the PC to PE ratio was significantly decreased in patients with NASH^39^ and could lead to necrosis of hepatocytes^51^,^52^. Next, we demonstrated that indeed membrane integrity was altered in the liver tissues with NASH. Therein, mass spectrometry imaging using ToF-SIMS imaging revealed lipid spreading in the hepatic parenchyma in NASH whereas lipids were mostly located in vesicles in NAFL. Such a spreading may result of loss of membrane integrity of hepatocytes in the context of NASH. Therefore, our study showed a potent loss of membrane integrity in NASH leading to a potent toxicity of the lipids released in the hepatic parenchyma, thus favoring the progression of the pathology.

By using an original approach combining the five fatty acids from the signature, toxicity of lipids accumulated in NASH was demonstrated on the human hepatoma cell line HepG2 and HPH. Although each of the five fatty acids studied showed toxicity individually^12^,^13^,^51^, the combination corresponding to the lipid composition observed in NASH based on our lipidomic analysis exhibited a much higher toxicity compared to those combination related to the composition observed in normal liver or NAFL. Our results suggested that lipotoxicity was not only related to the amount of lipids but also to their specific composition and proportion. It should be noted that the range of concentration tested from 50 μM to 1000 μM were lower than the concentration of these lipids estimated by lipidomics in the liver. This strengthened the idea that these five lipids at least could be highly toxic when in contact with neighboring hepatocytes.

In conclusion, we clearly characterized a specific and sensitive lipid signature, universal for all patients with NASH. This study highlighted dysregulations of the metabolic pathway involved in the synthesis of fatty acids and eicosanoid precursors. In particular, our study positioned ELOVL6 and FADS1 as major players in the progression to NASH. Finally, the current study suggested also a direct role of lipids accumulated in NASH in the progression of the pathology by their toxicity. This opens new avenues for further development of early diagnosis and therapeutic approaches.

## Materials and Methods

### Study Cohort

A total of 68 patients were enrolled in this study selected from two hospital centers (Paul Brousse Hospital, n = 61 and L’Archet Hospital, n = 7). A pathologist expert (CG) reviewed all liver biopsies. Seven patients had a normal liver forming the control group (hepatic steatosis <5%). Fifty four patients have been recorded as affected with NAFLD. To differentiate NAFL and NASH, a histological discrimination was made based on a separate system of scoring the features of NAFLD called the NAFLD Activity Score (NAS)^8^. By definition, a NAS < 5 represents NAFL and a NAS ≥ 5 represents NASH^8^,^42^. Clinical and biological data on general status, metabolic syndrome and liver function were retrospectively recorded. Exclusion criteria were liver diseases such as viral hepatitis B, viral hepatitis C, primary biliary cirrhosis, sclerosing cholangitis, autoimmune hepatitis, hemochromatosis, Wilson’s disease, α1-antitrypsin deficiency, drug-induced liver disease and alcohol consumption more than 20 g/day for women and 30 g/day for men. The institutional review board of each hospital (Paul Brousse Hospital through Centre des ressources biologiques Paris-Sud and L’ Archet Hospital) approved the study and written informed consent was obtained from all patients. Access to this material and all experiments were performed in accordance with the relevant guidelines and regulation of the French ethical laws.

### Animal Models

Male C57Bl/6J mice were fed on chow diet, HFD and MCDD. Mice fed a HFD and MCDD developed NAFL and NASH, respectively as described in our previous publication^18^. A total of 20 animals underwent chow diet (n = 10, Teklad Rodent Diet no. 5053; 5% kcal from fat; 3.1 kcal/g), HFD (n = 5; 15 weeks on diet, Research Diet D12492i; 60% kcal from fat; 5.24 kcal/g) and MCD diet (n = 5; 5 weeks on diet, TekladRef# TD.90262). Mice were housed at room temperature (22–24 °C) with a 12-hour light/12-hour dark cycle. Food and water were provided *ad libitum*. Animal protocols were accepted by the Institutional Animal Care and Use Committee in Main (Jackson Laboratories) and by the “Comité d’Ethique pour l’Expérimentation Animale” registered to the “Comité National de Réflexion Ethique sur l’Expérimentation Animale 05” (Protocol # Ce5/2012/075). All experiments were performed in accordance with the relevant guidelines and regulation of each country.

### Real-time quantitative PCR of genes involved in lipid metabolism

Total RNA was extracted from frozen liver biopsies using RNA-STAT 60 reagent (AMS Biotechnology Europe LTD). Quantity and quality of RNA were assessed using NanoDrop^®^-ND1000 (Thermo Scientific). cDNAs were generated by using the RivertAid^®^ First Strand cDNA Synthesis (Thermo Scientific), and Syber Green from FastStart Essential DNA Green Master mixes (Roche, Life Science) were used to quantify hepatic mRNA levels with specific primers of each gene described in Supplemental Table S1.

Q-RT-PCR was performed using LightCycler^®^ 96 Instrument (Roche, Life Science). Gene expression levels were normalized to actin RNA levels and data analyzed with LightCycler^®^ 96 SW 1.1 software (Roche, Life Science). For each sample, the gene to actin ratio was calculated based on an arbitrary value of copies determined by the standard curve for each gene, as previously described^53^.

### Activity indexes of desaturases and elongases

As standard method to evaluate fatty acid-synthetizing enzyme activities, assay measurement of the rate of radiolabeled precursor FA to their respective products is used *in vitro* and *in vivo*, but for practical and ethical reasons is not possible in human studies. Therefore product-to-precursor ratio as surrogate measure to estimate desaturase and elongase activities is assessed in this study as it has been extensively used in different other studies before^17^,^21^,^26^,^54^,^55^. Using the comprehensive lipid analysis by mass spectrometry data from human and mouse livers we assessed activity indexes of desaturases and elongases belonging to the long chain and very long chain saturated, monounsaturated and polyunsaturated of fatty acid synthesis pathway as summarized in Fig. 3.

### Isolation and primary culture of human hepatocytes

Normal liver tissue was obtained from adult patients undergoing partial hepatectomy at Saint Antoine Hospital (generous gift from Dr. Filomena Conti and Pr. Yvon Calmus, Paris, France). The first donor was a 63 years old woman treated for liver metastasis for colorectal adenocarcinoma. The second donor was a 36 years old female treated for hepatocellular carcinoma developed on normal liver. The third patient is a 65 years old man treated for liver metastasis of pancreatic cancer. Experimental procedures were performed in accordance with French laws and regulations. Human primary hepatocytes isolation was made based on previous protocol^56^,^57^. Briefly, immediately after hepatectomy liver resection specimen was stored in Celsior solution (IMTIX-SangStat), followed by a 2-steps perfusion method, less than 3 h after resection. Visible vessels were first perfused with Liver Perfusion Medium (Invitrogen) at 37 °C to eliminate blood cells. A second perfusion then was performed with collagenase- and dispase-containing Liver Digest Medium (Invitrogen) at 37 °C, at constant flow rate until the tissue was fully digested. Liver fragments were shaken gently in Hepatocyte Wash Medium (Invitrogen) to free loose cells, and then were filtered before centrifugation. The fibroblast- and Küpffer cell–containing supernatant was discarded, and hepatocytes were washed a second time before assessing viability by trypan blue dye exclusion. Cells were re-suspended in complete hepatocyte medium and seeded at a density of 5 × 10^5^ viable cells per well onto 96-well plates that had been pre-coated with a solution type I collagen from calf skin between 1 and 10 hours before plaiting cells. The medium was replaced 16–20 hours later with fresh complete hepatocyte medium supplemented with 1 mol/L hydrocortisone hemi-succinate 56,57 and 100 units/mL penicillin, and 100 g/mL streptomycin^56^,^57^. All cell cultures were maintained at 37 °C in a 5% CO_2_ atmosphere.

### Cell culture

HepG2 cells, derived from differentiated human hepatoblastoma^51^, were obtained from ATCC (Manassas, VA). Cells were cultured in DMEM containing 10% (v/v) FBS, 100 units/mL penicillin, and 100 g/mL streptomycin. The medium was changed 12 h before treatment. All cell cultures were maintained at 37 °C in a 5% CO_2_ atmosphere.

### Lipids preparation for treatments

Myristic acid (C14:0), palmitic acid (C16:0), palmitoleic acid (C16:1n-7), vaccenic acid (C18:1n-7) and oleic acid (C18:1n-9) were all obtained from Sigma (Ref# M3128, 43051, S4751, P9417, V0384, O1257, respectively). Fatty acids were dissolved in absolute ethanol at a concentration of 40 mM stock solutions, sonicated 15 min and then warmed at 70 °C for 15 min, for complete dissolution. FA solutions were filtered through a 0.22 μm filter before use and stored at −20 °C. Then, FA were dissolved in bovine serum albumin (10% BSA, Sigma) at the final ratio 1/10 (v/v) in William’s E plus Glutamax™ medium or in OptiMEM™ (Gybco, Invitrogen) and warmed again at 55 °C for 10 min before use^58^. The final concentration of ethanol did not exceed 1%. Myristic acid (C14:0), palmitic acid (C16:0), stearic acid (C18:0) palmitoleic acid (C16:1n-7), vaccenic acid (C18:1n-7) and oleic acid (C18:1n-9) were diluted individually or mixed in bovine serum albumin and ethanol to obtain 50, 100, 250, 500 and 1000 μM at the final concentration. Three mixtures so-called Control, NAFL2/3 and NASH were prepared based on the percentage of myristic acid, palmitic acid, palmitoleic acid, vaccenic acid and oleic acid found in liver of patients (Table 2). HepG2 cells or human primary hepatocytes (HPH) were treated with each lipid individually or with the lipid mix during 24 h. Lipotoxicity was assessed by the content of total ATP into the cells (CellTiter-Glo^®^ Luminescent Cell Viability Assay, Promega, France).

### Lipid Profiling by Mass Spectrometry Analysis

Liver biopsies (5–10 mg) were homogenized in 2 ml of methanol/EGTA (2:1 v/v) with FAST-PREP (MP Biochemicals) tissue lyser for further lipid analyses. Also, the equivalent of 0.5 mg of tissues was evaporated. The dry pellets were dissolved in 0.25 ml of NaOH (0.1 M) overnight and proteins were measured with the Bio-Rad assay. The quantification of the lipids is expressed in nmol/mg of total proteins.

Briefly, lipids were extracted from liver tissues according to Bligh and Dyer^59^ in dichloromethane/methanol/water (2.5:2.5:2.1, v/v/v), in the presence of the internal standards (stigmasterol, cholesteryl heptadecanoate, glyceryl trinonadecanoate) to quantify neutral lipids. Dichloromethane phase were evaporated to dryness, and the residue dissolved in 20 μl of ethyl acetate. 1 μl of the lipid extract was analyzed by gas-liquid chromatography on a FOCUS Thermo Electron system using a Zebron-1 Phenomenex fused silica capillary columns coupled to mass spectrometry according to previous publication^60^.

Phospholipids for relative quantification were extracted as neutral lipids but with 2% acetic acid in the presence of the internals standards (Cer(d18:1/15:0) 16 ng; PE(12:0/12:0) 180 ng; PC(13:0/13:0) 16 ng; SM(d18:1/12:0) 16 ng; PI(16:0/17:0) 30 ng; PS(12:0/12:0) 156.25 ng). After centrifugation the organic phase was collected and dried under azote, then dissolved in 50 μL of methanol. Lipids were separated using a Kinetex HILIC column with a mobile phase A of acetonitrile and B with 10 mM ammonium formate in water at pH 3.2 following a gradient and the injection volume was 5 μL. Sample solutions were analyzed using an Agilent 1290 UPLC system coupled to a G6460 triple quadrupole mass spectrometer (Agilent Technologies) and using MassHunter software (Agilent Technologies) for data acquisition and analysis. Data were treated using QqQ Quantitative (version B.05.00) and Qualitative analysis software (version B.04.00).

For Fatty Acid Methyl Ester (FAME) analysis, homogenate were extracted as neutral lipids in the presence of the internal standards glyceryl triheptadecanoate (2 μg) and transmethylated 1 h in boron trifluoride methanol solution 10% at 55 °C. After addition of water (1 ml) to the crude, FAMEs were extracted with hexane (3 ml), evaporated to dryness and dissolved in ethyl acetate (20 μl). FAME (1 μl) was analyzed by gas-liquid chromatography on a Clarus 600 Perkin Elmer system using Famewax RESTEK fused silica capillary columns.

### Time-of-Flight Secondary Ion Mass Spectrometry Imaging

#### Tissue preparation

A subset group of patients with the four grades of liver steatosis (3 patients per group) and a group of patients with NASH (4 patients) underwent for ToF-SIMS imaging analyses.

Briefly, each sample was cut at −20 °C with a CM3050-S cryostat (Leica Microsystèmes SAS, France). Tissue sections of 10 μm thickness were deposited on a gold coated glass slide (Mirr IR^®^, Kevley Technologies, OI, US). Before analysis, tissue sections were placed under vacuum at a pressure of a few hPa during 10 min in order to eliminate water. Before analysis, tissue samples were examined and optical images were recorded with a microscope (Olympus BX 51, Olympus France, SAS, Rungis, France) equipped with a ColorView I camera monitored by Cell^B^ software (Soft Imaging System GmbH, Münster, Germany). No further sample preparation was required before introduction in the mass spectrometer.

#### TOF-SIMS imaging acquisition

A TOF-SIMS IV mass spectrometer (ION-TOF GmbH, Münster, Germany), equipped with a Liquid Metal Ion Gun (LMIG) filled with bismuth and allowing delivery of Bi_3_^+^ cluster ion beam was used to localized lipids of interest directly on the liver tissues as previously described^61^.

Briefly, a set of images was acquired without sample stage movement, just by resting the primary ion beam, with a field of view of 500 μm × 500 μm. For these images, the number of pixels was chosen as 256 × 256 to obtain a ~2 μm pixel size. Under these conditions the flow was fixed to 3 × 10^11^ ions/cm^2^ for all the acquisitions, allowing acquisition time of about 10 minutes. Each area was scanned twice in order to record both positive and negative ion images.

Due to the very low initial kinetic energy distribution of the secondary ions, the relationship between the time-of-flight and the square root of *m*/*z* is always linear over the whole mass range. Consequently, the mass calibration was made with H^−^, C^−^, CH^−^, CH_2_^−^, CH_3_^−^, C_2_^−^, C_3_^−^, and C_4_H^−^ ions for the negative ion mode, and H^+^, H_2_^+^, H_3_^+^, C^+^, CH^+^, CH_2_^+^, CH_3_^+^ and C_2_H_5_^+^ for the positive ion mode, respectively. To refine the mass calibration, ion peaks of cholesterol and vitamin E were used in positive ion mode, and fatty acid carboxylate ions in negative ion mode based on previous studies reported on biological samples^61^,^62^,^63^,^64^.

Data processing was achieved using Surface Lab 6.2 software (ION-TOF GmbH, Münster, Germany). This software allows extraction of ion spectra and images from the raw data. In order to compare the relative intensity of species in the first set of experiments, a normalization of their respective mass spectrum intensities had to be performed: the intensity of the mass spectrum from each stage scan was normalized against the area of the smallest one, given that all the data had been acquired under the same experimental conditions^61^,^65^,^66^,^67^.

#### ToF-SIMS data analysis

We selected 3 patients in each group of control, NAFL1, NAFL2 and NAFL3 as well as 4 patients with NASH. The sum of the diacylglycerols (DGs) served to localize the lipid droplets into the liver tissue. C14:0, C16:0, C16:1, C18:0, C18:1, C18:2 and C20:4 were investigated using ToF-SIMS procedure in different areas of the liver. The size of each patch was 500 μm × 500 μm^61^.

### Statistical Analysis

Lipidomic data of patients with NASH from Paul Brousse Hospital were used as learning dataset (NASH_Lds) whereas patients with NASH L’Archet Hospital were used as validation dataset (NASH_Vds).

In order to be sure that the number of patients per group is acceptable for further statistical analyses, we performed a statistical parametric test, MANOVA (multivariate analysis of variance), using GPower software (version 3.1.9.2) and including the following parameters such as: effect size (f^2^_(v)_ = 0.25), α = 0.05, power (1-β = 0.95), and the number of groups (n = 6).

All calculations were performed using R v.3.3.1 software^68^. To analyze the homogeneity of the groups of patients with NAFL, recursive partitioning and regression trees (“*rpart*”) approach was used to build a regression tree based on the predict values from the lipidomic data leading to obtain a classification analysis and a regression tree (CART). CART was applied on lipid families such as total TG, total DG, Total cholesterol, Total CE, Total SFA, Total USFA, Total MUFA, Total PUFA, Total PC, Total PE, Total PI, Total PS, Total Cer and Total SM (Supplemental Fig. S1).

In order to identify the specific dependent variables (lipids) that contributed to the significant overall effect (between different NAFL groups and between NAFL and NASH groups), a random forests (RF) analysis was used with the following R packages “*randomForest*” and “*varSelRF*” leading to obtain a narrow numbers of markers, as we published recently^18^. Briefly, RF consisted of a collection of tree predictors where each tree depended on the value of a random vector of measured variables sampled independently and with the same distribution for all trees in the forest. RF classified a case by assigning the input vector of variables to each tree of the forest. Each tree gave a classification, *i.e*. a classis voted, and the forest chose the class with the most votes from all the trees in the forest^25^,^69^. RF analysis was an effective tool in prediction without over-fitting and multiclass classification^25^,^69^,^70^,^71^. As in many statistical analyses leading to a lot of variables and few groups (as we face here: 104 lipids and 5 groups of patients), a crucial problem was variables not significantly relevant to explain the analyzed phenomenon (*i.e*. occurrence of NASH) and missing values, but could create a random noise which hided the main effects and the relevant predictors^25^. Thus to determine the most discriminant lipids, RF were applied using “*randomForest*” package in R. To determine the best number of predictors (*mtry*) was used for each split of the tree and *tune RF* function was used to determine the lowest *mtry* to the lowest out-of-bag (OOB) error data that was used to get a running unbiased estimate of the classification error as trees were added to the forest melding to determine the confusion matrix (Supplemental Fig. S2a and b). Also *ntree* (number of trees to be built) was set up at 1040 corresponding to the number of the columns (variable) of the matrix multiply by ten. During the analysis, the mean decreased accuracy (MDA) and the mean decreased Giny (MDG) were determined. MDA was determined during the OOB error calculation phase and lipids with a large MDA were more important for classification of the data (Supplemental Fig. S2c and d). In addition, MDG that was a measure of how each variable contributes to the homogeneity of the nodes and leaves in the resulting RF was assessed. Lipids that resulted in nodes with higher purity had a higher MDG (Supplemental Fig. S2d).

Principal component analysis, 95% confident ellipse centre to the mean and lipids of interest were computed using “*FactoMinR*” package. The global p-value was calculated using the critical probability associated with the F- test of the analysis of variance along the axes of the first and the second dimensions (α = 0.05). Receiver operating characteristic (ROC) curve was analysed using “*pROC*” and “*Epi*” packages and compared control, NAFL1, NAFL2 and NAFL3 versus NASH patients from Paul Brousse and L’Archet Hospitals based on the 32 lipids identified by random forests. Boxplots were drawn using “*ggplot2*” and “*beeswarm*” packages. “*gplots*” and “*RColorBrewer*” packages were used for graphics.

Individual variables among the different groups of patients were shown as boxplot or barplot (mean ± standard error of the mean or SEM) and tested with analysis of variance (ANOVA-test) followed by unpaired *t*-test. Kruskal-Wallis rank sum test was used to compare gender repartition between the 6 groups of patients. Type I error-set was 5%.

### Ethics approval

The institutional review board of each hospital and ethic committee (Paul Brousse Hospital Centre des resources biologiques Paris-Sud and L’Archet Hospital) approved the study and written informed consent was obtained from all patients. Access to this material was in agreement with French ethical laws.

Animal protocols were accepted by the Institutional Animal Care and Use Committee (IACUC) in Main (Jackson Laboratories) and by the “Comité d’Ethique pour l’Expérimentation Animale” registered to the “Comité National de Réflexion Ethique sur l’Expérimentation Animale 05” (Protocol # Ce5/2012/075, Paris, France). In accordance to the animal welfare and in the aim to minimize the number of animals, we used data and samples from our previous publication^18^.

## Additional Information

**How to cite this article:** Chiappini, F. *et al*. Metabolism dysregulation induces a specific lipid signature of nonalcoholic steatohepatitis in patients. *Sci. Rep.* **7**, 46658; doi: 10.1038/srep46658 (2017).

**Publisher's note:** Springer Nature remains neutral with regard to jurisdictional claims in published maps and institutional affiliations.

## Supplementary Material

Supplementary Dataset 2

Supplementary Dataset 1

Supplementary Information

## Figures and Tables

**Table 1:** Characteristics of the study population.

	Control n = 7	NAFL1 n = 9	NAFL2 n = 12	NAFL3 n = 18	NASH_Lds n = 15	NASH_Vds n = 7
Gender F/M	5/2	6/3	6/6	4/14	10/5	7/0^#^
Age (years)	36.4 ± 5.2*	49.7 ± 5.7	60.1 ± 3.5	61.1 ± 3.6	54.7 ± 2.5	54.6 ± 3.9
BMI (kg/m^2^)	21.0 ± 1.0	22.1 ± 1.1	26.1 ± 1.3*	27.7 ± 0.8*	31.5 ± 1.6*	42.9 ± 1.8*^†^
Fasting glucose (mmol/L)	6.1 ± 0.4	6.9 ± 1.3	5.1 ± 0.3	6.1 ± 0.4	7.2 ± 0.6	5.1 ± 0.2
AST (normal range 0–65 IU/L)	27.4 ± 2.5	18.5 ± 3.7	82.2 ± 52.1	29.2 ± 2.9	33.8 ± 3.1	35.4 ± 3.2
ALT (normal range 0–65 IU/L)	29.6 ± 4.2	19.0 ± 3.6	59.4 ± 24.2	34.7 ± 4.9	40.6 ± 5.1	49.9 ± 5.3
γ-GT (IU/L)	106.0 ± 41.3	50.4 ± 6.3	193.1 ± 93.6	83.5 ± 22.6	129.2 ± 29.8	36.7 ± 6.5*^†^
Alkaline phosphatase (IU/L)	132.9 ± 42.8	87.8 ± 7.3	182.5 ± 68.0	91.0 ± 8.3	114.3 ± 25.7	69.9 ± 6.4
Total bilirubin (mgL)	14.3 ± 2.2	12.6 ± 2.4	11.6 ± 2.4	13.9 ± 1.8	10.4 ± 1.2	8.2 ± 1.7
Albumin (g/L)	37.4 ± 1.1	39.4 ± 0.9	38.5 ± 0.8	35.7 ± 0.8	37.8 ± 0.7	43.6 ± 1.4
Platelets (giga/L)	271.9 ± 40.5	252.6 ± 21.8	267.5 ± 32.6	224.2 ± 16.7	230.8 ± 26.3	279.0 ± 11.3
Steatosis grade (%)	0	15 ± 3	32 ± 8	53 ± 5	58 ± 6	81 ± 3
NAFLD activity score (NAS)	0.6 ± 0.2	1.3 ± 0.2	1.4 ± 0.2	2.5 ± 0.2	5.9 ± 0.2*	5.0 ± 0*
Fibrosis stage, 0/1a/1b/1c/2/3						
n patients	5/2/0/0/0/0	6/2/1/0/0/0	7/3/1/0/1/0	5/6/5/0/2/0	0/6/3/1/3/2	0/5/1/1/0/0
(%),	(71/19/0/0/0/0)	(67/22/11/0/0/0)	(59/25/8/0/8/0)	(28/33/28/0/11/0)	(0/40/20/7/20/13)	(0/72/14/14/0/0)

All patients are Caucasian. Data are expressed as mean ± SEM. The different groups were compared using ANOVA-test. *p < 0.05 *versus* Control (unpaired *t*-test); ^†^p < 0.01 *versus* NASH_Lds (unpaired *t*-test). Genders repartition between the 5 groups of patients with p = 0.05317 and # between 6 groups of patients p = 0.008243 by Kruskal-Wallis rank sum test. ALT, alanine aminotransferase; AST, aspartate aminotransferase; BMI, body mass index; F: female; γ-GT, gamma-glutamyl transferase; m: male; NAFL, nonalcoholic fatty liver; NAFLD, nonalcoholic fatty liver disease; NASH, nonalcoholic steatohepatitis. Control, NAFL1, NAFL2, NAFL3 and NASH_Lds groups of patients selected at Paul Brousse Hospital (Villejuif, France). NASH_Vds are patients from L’Archet Hospital (Nice, France). NASH_Lds: learning dataset cohort; NASH_Vds: validation dataset cohort.

**Table 2:** Concentrations and proportions of the 5 discriminant fatty acids in human livers.

	Patients	Myristic acid	Palmitic acid	Palmitoleic acid	Vaccenic acid	Oleic acid	Total
FA concentration* in human livers expressed in nmol/mg of proteins	**Control**	0	138.04	10.54	14.32	52.52	215.41
	**NAFL2 + 3**	14.24	558.87	56.37	35.87	393.44	1058.81
	**NASH**	56.95	1044.95	180.52	70.89	976.53	2329.34
**Percentage of FA in liver tissues**	**Control**	0	64	5	24	7	100
	**NAFL2 + 3**	1.5	53	5.5	3	37	100
	**NASH**	2.5	45	7.5	3	42	100

FA: fatty acid; NAFL: nonalcoholic fatty liver; NASH: nonalcoholic steatohepatitis. *Mean of each lipid assessed by lipidomic analysis found in each group of patients: Control (n = 7), NAFL 2 and NAFL3 (n = 30), NASH (n = 22).
