# Supplementary material for: Metabolism dysregulation induces a specific lipid signature of nonalcoholic steatohepatitis in patients
Source: Sci Rep. 2017 Apr 24;7:46658. doi: 10.1038/srep46658 (PMC5402394; doi:10.1038/srep46658)
Supplement: Supplementary Information [file srep46658-s3.pdf]

Metabolism dysregulation induces a specific lipid signature of nonalcoholic steatohepatitis in patients

Franck Chiappini, Audrey Coilly, Hanane Kadar, Philippe Gual, Albert Tran, Christophe Desterke, Didier Samuel, Jean-Charles Duclos-Vallée, David Touboul, Justine Bertrand-Michel Alain Brunelle, Catherine Guettier François Le Naour

Supplementary information (Online)

Supplemental Figure S1

Supplemental Figure S2

Supplemental Figure S3

Supplemental Figure S4

Supplemental Figure S5

Supplemental Figure S6

Supplemental Table S1

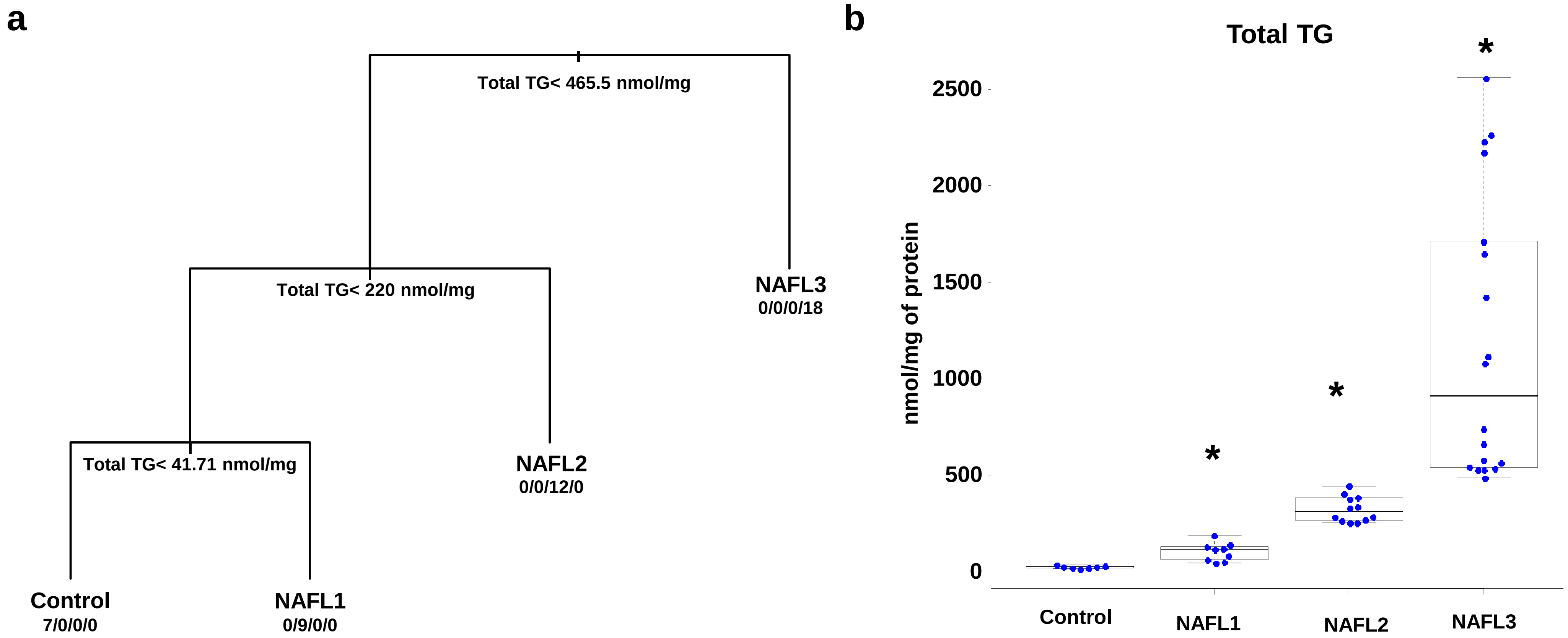

Supplemental Figure S1. Total hepatic triglycerides clustered the four groups of patients with different grades of NAFL based on cluster analysis regression tree (CART). (a) Data are represented as a regression tree where each node of the tree corresponds to the total triglycerides (TG): Ranges of Total TG liver content determine the four groups of patients. **(b)** Total TG content from human liver biopsies in clustered patients based on the CART analysis from (A). Data are represented as boxplot. *p<0.05, by unpaired *t*-test compared to each other groups after ANOVA analysis. Control n=7; NAFL1 n=9; NAFL2 n=12; NAFL3 n=18. NAFL: nonalcoholic fatty liver.

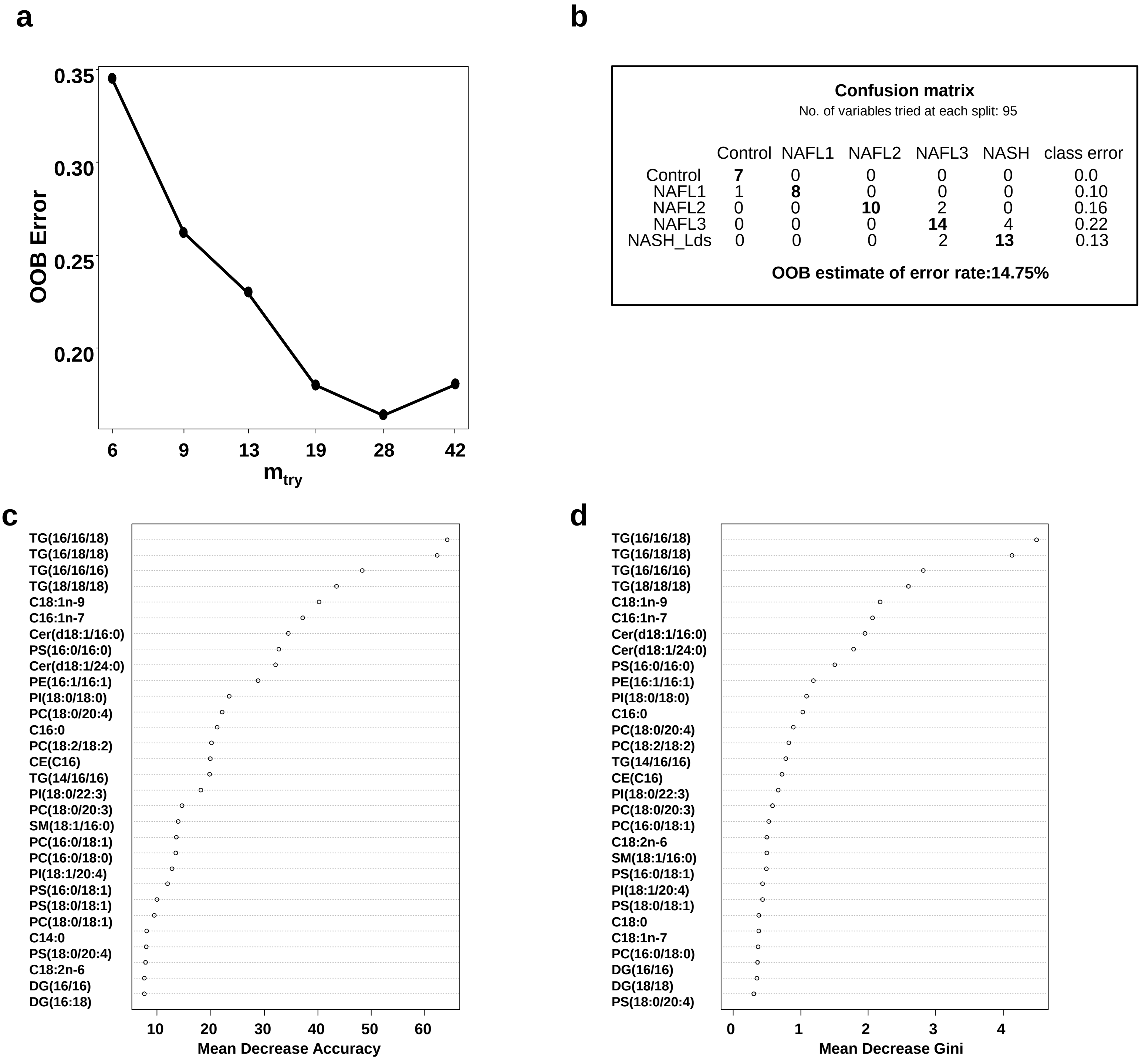

Supplemental Figure S2. Some of lipids selected after random forests analysis clustered NASH patients compared to other groups of patients with different grades of liver steatosis. (a) Determination of the best m_{try} (i.e. the best number of randomly preselected splitting variables) associated to the minimum out-of-bag (OOB) estimate of error rate. **(b)** associated to the confusion matrix The 30 first lipids sorted by random forests analysis and classified based on their **(c)** mean decrease accuracy and **(d)** mean decrease Gini indexes to discriminate NASH patients versus the 4 other groups. Control n=7; NAFL1 n=9; NAFL2 n=12; NAFL3 n=18; NASH_Lds n=15. NAFL: nonalcoholic fatty liver disease; NASH_Lds: nonalcoholic steatohepatitis learning dataset.

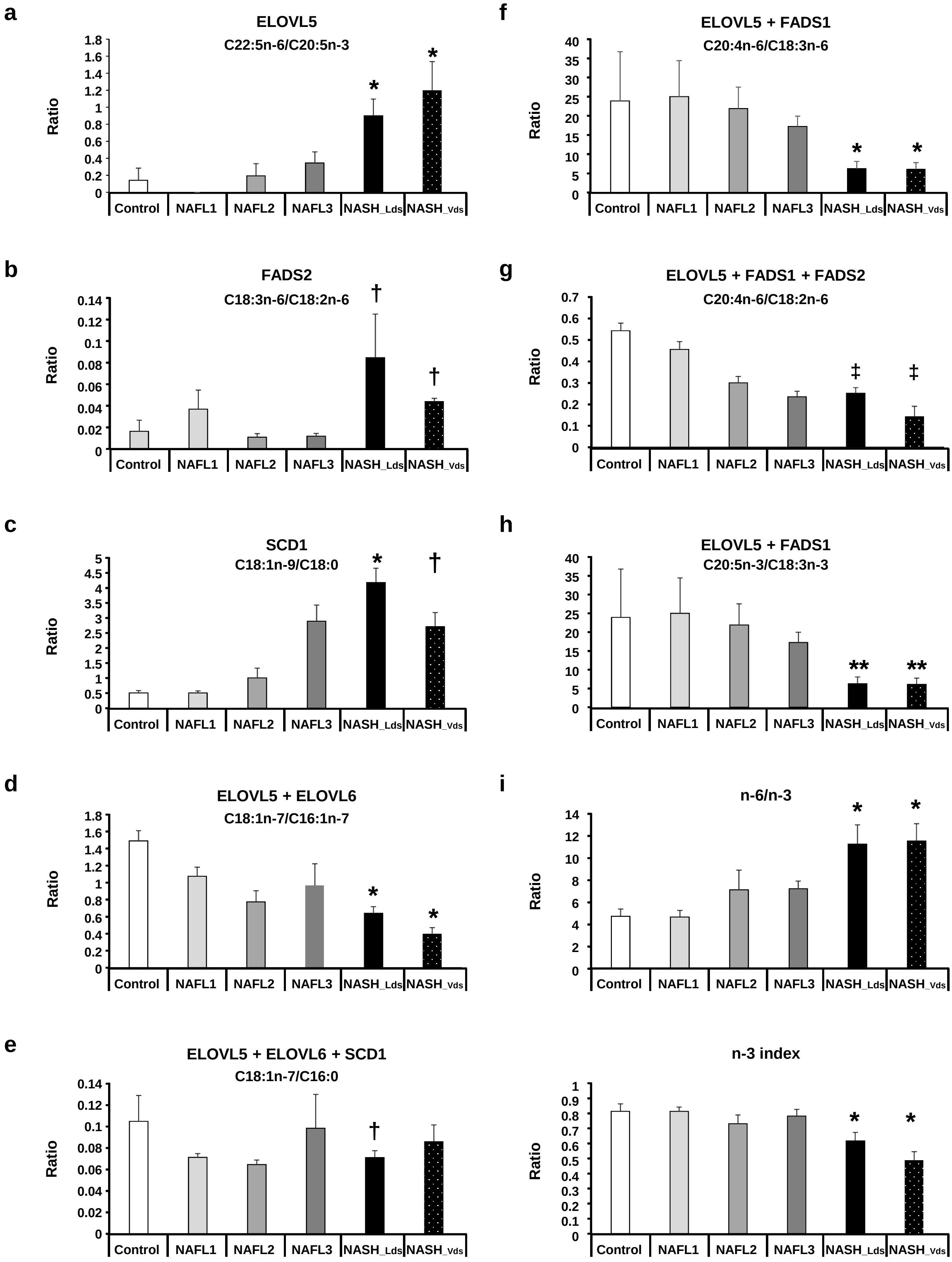

Supplemental Figure S3. Decrease in FADS1 and ELVOL6 index activities in NASH patients. (a) Evaluation of ELOVL5 activity using c22:5n-6 to c20:5n-3; **(b)** evaluation of FADS2 activity using c18:3n-6 to c18:2n-6 ratio. **(c)** evaluation of SCD1 using c18:1n-9 to c18:0 ratio **(d)** both ELOVL5 and ELOVL6 activities using c18:1n-7 to c16:1n-7; **(e)** global ELOVL5. ELOVL6 and SCD1 activities using c18:1n-7 to c16:0 ratio; **(f)** both ELOVL5 and FADS1 activities using c20:4n-6 to c18:3n-6 ratio. **(g)** Global ELOVL5. FADS1 and FADS2 activities using 20:4n-6 to c18:2n-6 ratio towards the n-6 pathway, and **(h)** global ELOVL5 and FADS1 activities using 20:5n-3 to c18:3n-3 ratio towards the n-3 pathway. **(i)** Hepatic n-6 to n-3 ratio and n-3 index of the different study groups. in order to evaluate enzyme activities. a ratio between product-to-precursor of each reaction has been used as described before**.** Data are shown as means ± SEM. *p<0.05 and **p<0.01 by unpaired *t*-test compared to each other groups ‡<0.05 by unpaired *t*-test compared to Control. NAFL1 and NAFL2. +<0.05 by unpaired *t*-test compared to Control and NAFL1. †<0.05 by unpaired *t*-test compared to Control. NAFL2 and NAFL3 after ANOVA analysis. Control n= 7; NAFL1 n=9; NAFL2 n=12; NAFL3 n=18; NASH_Lds n= 15, NASH_Vds n= 7. ELOVL: elongase of very long chain fatty acid; FADS: fatty acid desaturase; NAFL: nonalcoholic fatty liver; NASH: nonalcoholic steatohepatitis; NASH_Lds: learning dataset; NASH_Vds: validation dataset; SCD: stearoyl-CoA desaturase.

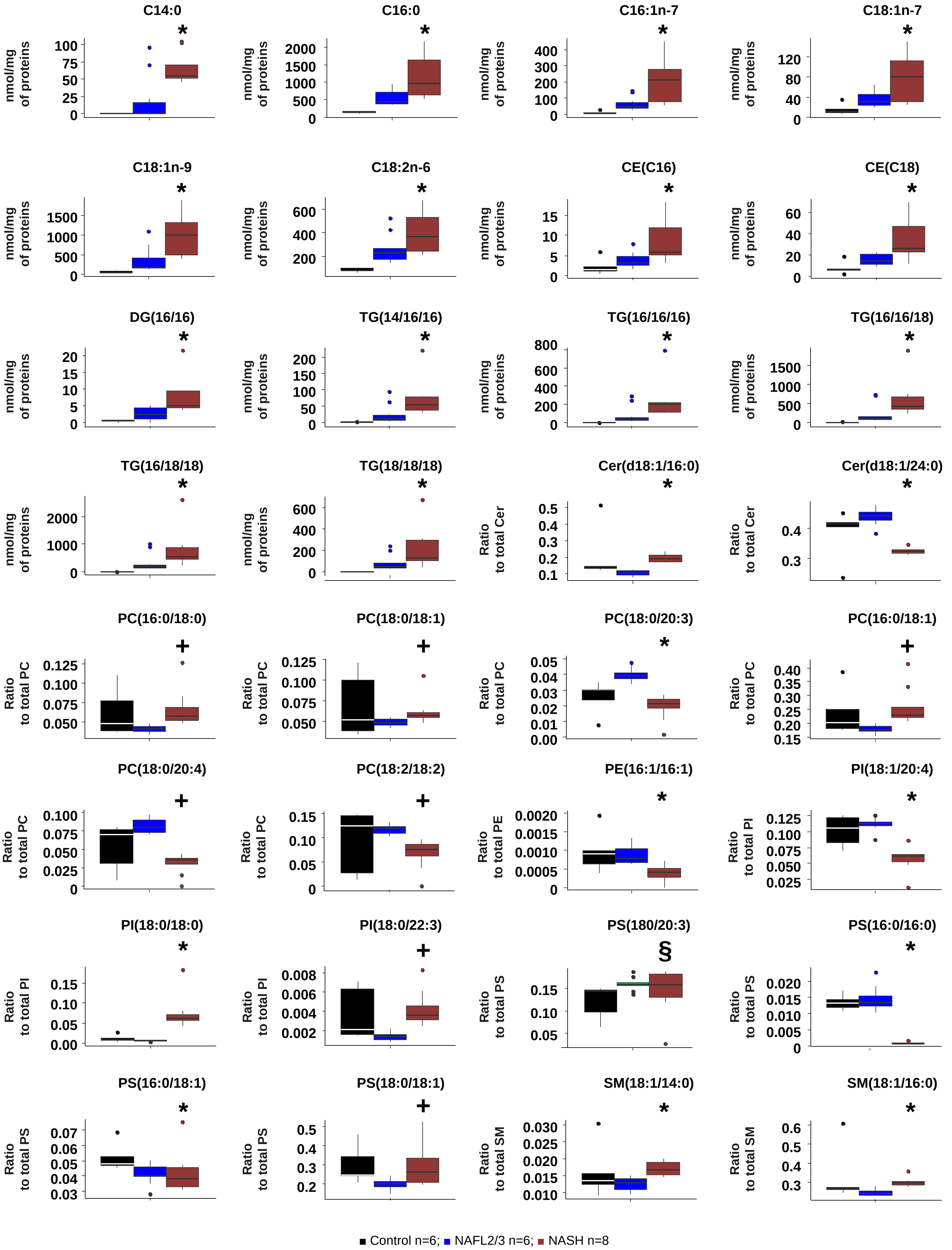

Supplemental Figure S4. Hepatic levels of the 32 lipids discriminating NASH group based on random forests analysis from liver biopsies used to look at metabolic gene expression levels involved in *de novo* lipid synthesis. (A) 14 fatty acids including free fatty acids, cholesteryl ester (CE), diglycerides (DG) and triglycerides (TG) “up-regulated” in NASH group. Relative abundance of phospholipids “up-regulated” (n=9) or **“**down-regulated” (n=9) in NASH group. Data are represented as boxplot. *p<0.05, by unpaired *t*-test compared to Control and NAFL2/3 groups. +p<0.05 by unpaired *t*-test compared NAFL2/3 group. §p<0.05 by unpaired *t*-test compared to Control group. Unpaired *t*-test was done after ANOVA test. ■ Control n=6; ■ NAFL2/3 n=6; ■ NASH n=8. NAFL2/3 group matched age, sex, grade od steatosis to NASH composed by patients from Paul Brousse and Nice hospitals. Cer: Ceramides; NAFL: nonalcoholic fatty liver; NASH: nonalcoholic steatohepatitis; PC: Phosphatidylcholines; PE: Phosphatidylethanolamines; PI: Phosphatidylinositols; PS: Phosphatidylserines; SM: Sphingomyelins

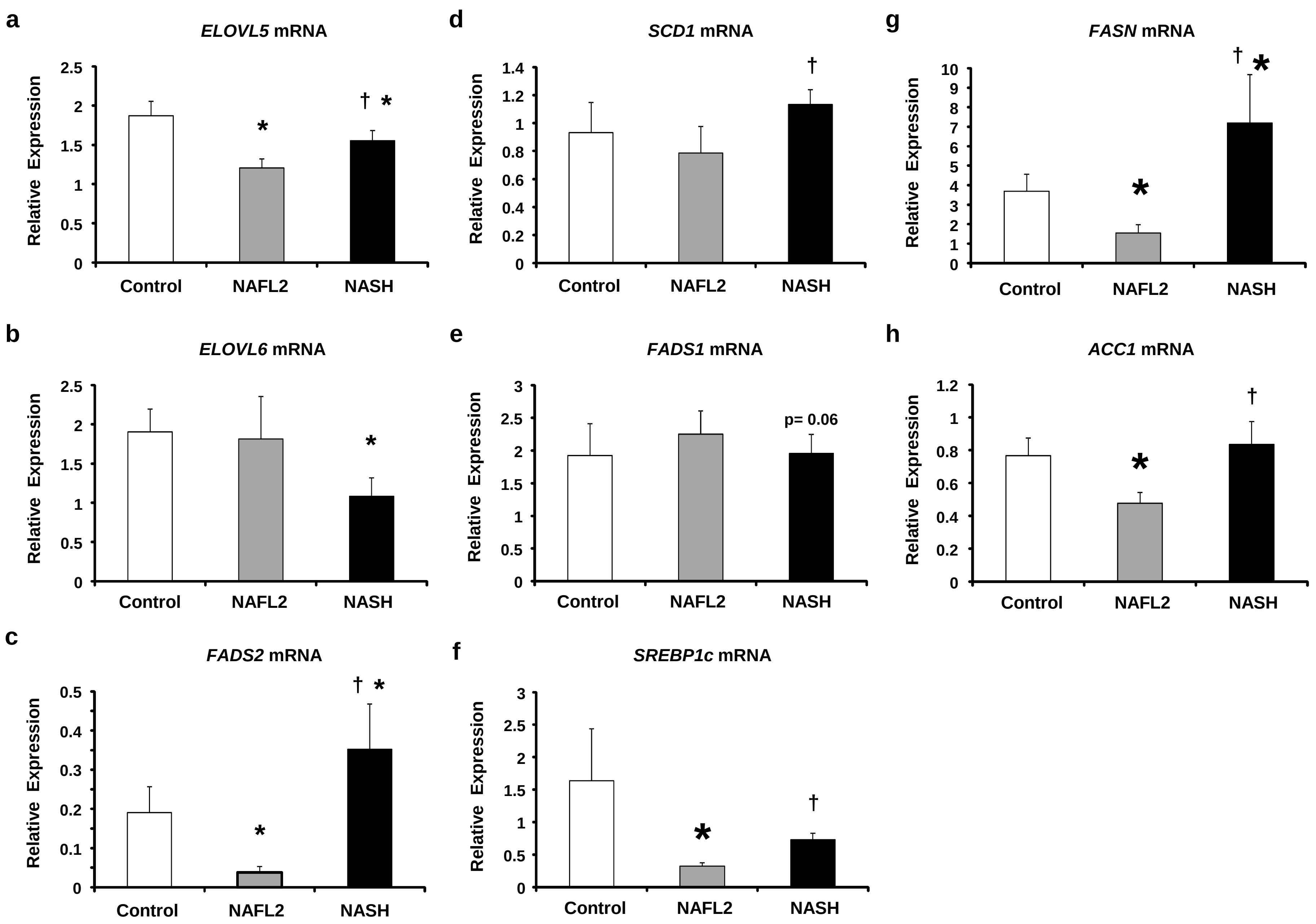

Supplemental Figure S5. Elongases and desaturases involved in LCPUFA synthesis are partially regulated at a transcriptional level. (a) *ELOVL5.* **(b)** *ELOVL6*. **(c)** *FADS2*. **(d)** *SCD1*. **(e)** *FADS1.* **(f)** *SREBP1c*. **(g)** *FASN and* **(h)** *ACC1* genes expression from human liver biopsies analyzed by RT-Q-PCR. Data are mean ± SEM. Control patients (n=6). NAFL2 patients (n=6) were matched to NASH patients (n=8).). NASH are matched to NAFL2 regarding to the total lipid content and no difference in age. gender and BMI between the two groups. *p≤ 0.05 compared to Control. †p≤0.05 compared to NAFL2 by unpaired *t*-test. ACC: Acetyl-CoA Carboxylase; ELOVL: elongase of very long-chain; FADS: fatty acid desaturase; FASN: fatty acid synthase; NAFL: nonalcoholic fatty liver; NASH: nonalcoholic steatohepatitis; SCD: stearoyl-CoA desaturase.

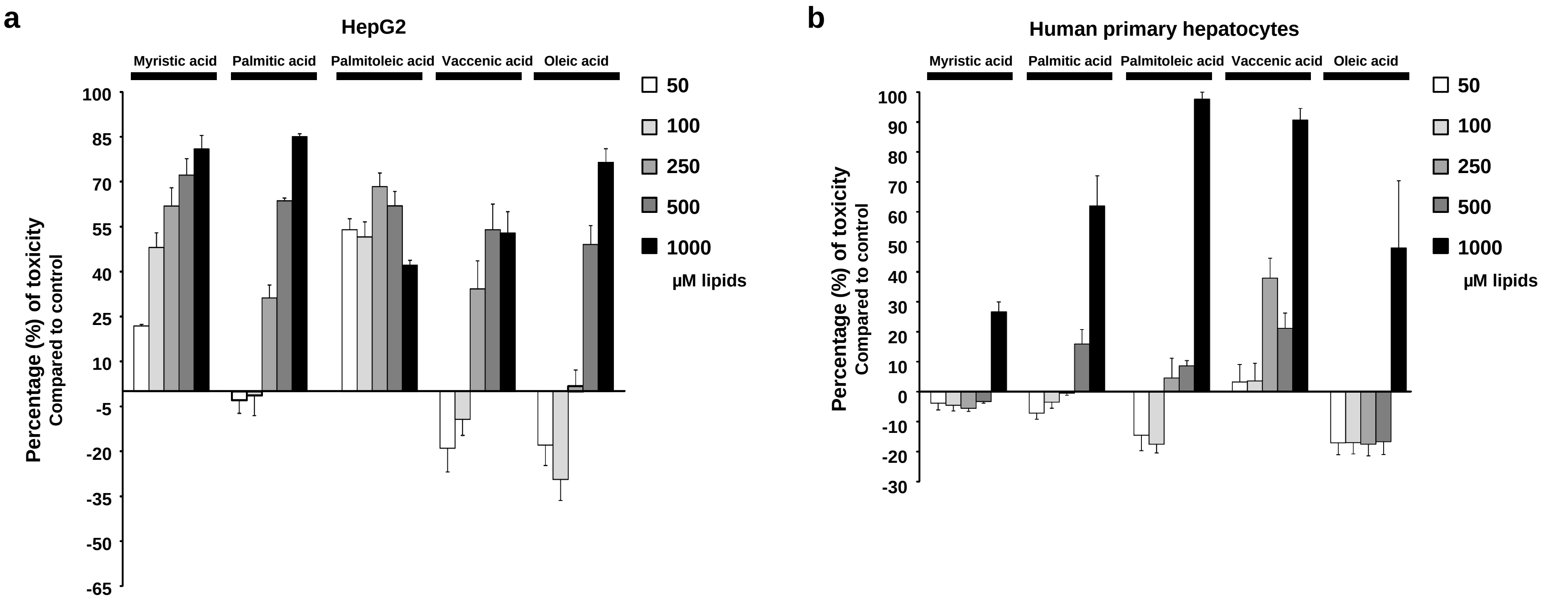

SupplementalFigure S6. Hepatic cells exhibit high toxicity after treatment with lipids from the NASH signature. Percentage of toxicity over control cells (non-treated) assessed by total ATP content into HepG2 human hepatoma cell line (left panel) and human primary hepatocytes (right panel). **(a)** HepG2 cell and **(b)** human primary hepatocytes treated with individual lipids at different concentrations. Two independent experiments were done. Data are mean ± SEM. *$p<0.05$ by unpaired *t*-test compared to Control Mix and NAFL Mix at the same concentration, after ANOVA analysis. NAFL: nonalcoholic fatty liver; NASH: nonalcoholic steatohepatitis.

Supplemental Table S1: List of primers used for Q-RT-PCR and implicated in hepatic human lipogenesis

Genes	Accession number	NCB	Primer Forward 5'-3'	Primer Reverse 5'-3'	TM
ELOVL6_human	AK027031	NM_024090	GCAAACACAAAACCCAAGGC	TGGCTTGCTTTTGTTCTCCC	58,99
ELOVL5_human	AF231981	NM_021814	GGACTCACACTGCTGTCTCT	GTTGTTCTTGCGCAGGATGA	59,1
ELOVL3_human	BC034344	NM_152310	AACCTCATTCCCCATAGCCC	AGCACACGGTTTGCTTTAGG	59,1
SCD1 (delta(9)-desaturase)_human	AF097514	NM_005063	TGAAAGCCAACAACTCTGCC	GCTGGACACTGAGCAAAGAC	59
FADS2 (delta(6)-desaturase)_human	AF126799	NM_004265	TTCCAAGGAGCAGAGAGGTG	CCCTATGAACCCCAAGAGCA	59
FADS1 (delta(5)-desaturase)_human	AF199596	NM_013402	TGCAATGTCCACAAGTCTGC	AGCTGCCCTGACTCCTTTAG	59
SREBP1c_human	AB373959	NM_004176	ACACAGCAACCAGAAACTCAAG	AGTGTGTCCTCCACCTCAGTCT	59
FASN_human	BC063242	NM_004104	CCCTCATCTCCCCACTCATC	CAGCGTCTTCCACACTATGC	59
ACC1_human	AY315627	NM_198834	TTGACTCCTCCATCAACCCC	AATTCCTCCCGCTCCTTCAA	59
Actin Beta_human	X00351	NM_001101	CATCCGCAAAGACCTGTACG	CCTGCTTGCTGATCCACATC	59

Primers were design based on the mRNA sequence found in Uniprot database and using Primer3 software and BLASTed (Basic Local Alignment Search Tool) on https://genome.ucsc.edu genomes

ACC: acetyl CoA carboxylase; ELOVL: elongase of very long chain fatty aid; FADS: fatty acid desaturase; FASN: fatty acid synthesis; SCD: stearoyl CoA desaturase; SREBP; sterol-regulated transcription factors.
